# LESSONS FROM FOUR DECADES IN AGING POLICY AT THE UNIVERSITY OF SOUTHERN CALIFORNIA: WHAT A LONG FINE TRIP IT’S BEEN

**DOI:** 10.1093/geroni/igad104.1775

**Published:** 2023-12-21

**Authors:** Kathleen Wilber, Maria Henke, Mireille Jacobson

**Affiliations:** University of Southern California, Los Angeles, California, United States; University of Southern California, Los Angeles, California, United States; University of Southern California, Los Angeles, California, United States

## Abstract

Aging policy has come a long way over the last four decades. Building on the history of leadership in policy and aging at the University of Southern California’s Leonard Davis School of Gerontology, we offer six lessons from the intersection of policy, practice, and research for researchers and for those interested in careers as policy gerontologists. Lessons include: 1) Researchers must communicate scientific truths clearly, humbly, often, relevantly, and broadly to a wide array of key stakeholders; 2) To continue to build the bench for the next generation of leaders, interested educators should convene to plan a Policy Leadership Program 2.0. 3) Researchers and practitioners should remember: If everything is going according to plan, something is wrong; 4) Those considering policy research, program development, or program evaluation should recognize that it can be funded by a variety of sources with different strengths and constraints; 5) Given a host of innovative ideas, perspectives, programs and services, in the aging policy space, evaluators and researchers will learn far more from those doing policy and practice work than they can ever hope to repay; 6) Progress is not linear nor does it consistently advance in the hoped for direction. It requires patience, persistence, and stamina.

